# Transcriptome/Degradome-Wide Discovery of MicroRNAs and Transcript Targets in Two *Paulownia australis* Genotypes

**DOI:** 10.1371/journal.pone.0106736

**Published:** 2014-09-08

**Authors:** Suyan Niu, Guoqiang Fan, Enkai Xu, Minjie Deng, Zhenli Zhao, Yanpeng Dong

**Affiliations:** 1 Institute of Paulownia, Henan Agricultural University, Zhengzhou, Henan, P.R. China; 2 College of Forestry, Henan Agricultural University, Zhengzhou, Henan, P.R. China; National Taiwan University, Taiwan

## Abstract

MicroRNAs (miRNAs) are involved in plant growth, development, and response to biotic and abiotic stresses. Most of the miRNAs that have been identified in model plants are well characterized, but till now, no reports have previously been published concerning miRNAs in *Paulownia australis*. In order to investigate miRNA-guided transcript target regulation in *P. australis*, small RNA libraries from two *P. australis* (diploids, PA2; and autotetraploids, PA4) genotypes were subjected to Solexa sequencing. As a result, 10,691,271 (PA2) and 10,712,733 (PA4) clean reads were obtained, and 45 conserved miRNAs belonging to 15 families, and 31 potential novel miRNAs candidates were identified. Compared with their expression levels in the PA2 plants, 26 miRNAs were up-regulated and 15 miRNAs were down-regulated in the PA4 plants. The relative expressions of 12 miRNAs were validated by quantitative real-time polymerase chain reaction. Using the degradome approach, 53 transcript targets were identified and annotated based on Gene Ontology and Kyoto Encyclopedia of Genes and Genomes pathway analysis. These targets were associated with development, stimulus response, metabolism, signaling transduction and biological regulation. Among them, 11 targets, including TCP transcription factors, auxin response factors, squamosa promoter-binding-like proteins, scarecrow-like proteins, L-type lectin-domain containing receptor kinases and zinc finger CCCH domain-containing protein, cleaved by four known miRNA family and two potentially novel miRNAs were found to be involved in regulating plant development, biotic and abiotic stresses. The findings will be helpful to facilitate studies on the functions of miRNAs and their transcript targets in *Paulownia.*

## Introduction

MicroRNAs (miRNAs), a class of endogenous 21–24 nucleotide (nt), single-stranded, non-coding RNAs, modulate the gene expression at the transcription and post-transcriptional levels [1], [2]. In plants, emerging data demonstrate that miRNAs play vital regulatory roles in a wide range of biological processes, including regulation of plant growth, development, signal transduction [3]–[6], and response to biotic and abiotic stresses via interactions with their specific target mRNAs [7]–[10]. Thereby, understanding of miRNAs functions in plant requires the recognition of their target genes. Initially, based on the either perfect or near-perfect sequence complementarity between miRNAs and their target mRNAs, the plant miRNA transcript targets have been identified via computational prediction [11], [12]. However, computational target prediction method is very challenging because of the existence of a higher mismatch in miRNA-target mRNA pairing [13]. With the development of high-throughput sequencing technology, recently, a transcriptome wide experimental method combining high-throughput miRNA profiling with degradome sequencing analysis, has been successfully developed to identify miRNA-directed mRNA cleavage at a large scale [14], [15]. Using this strategy, so many plant species have been studied, for instance, *Arabidopsis* [15], *Oryza sativa* [16], *maize* [17], *Brassica napus* [18], *Medicago truncatula* [19], *Fragaria ananassa* [20], *grapevine* [21], *Taxus* [22], *Populus trichocarpa* [23], *Populus euphratica* [24], *Trifoliate orange* [25], *Paulownia tomentosa* [26], and *Paulownia fortunei* [27]. These discoveries have triggered detailed biological studies on gene regulation with the result that the number of reported plant miRNAs and their transcript targets has been increasing rapidly.

*Paulownia* is a genus of deciduous tree species in the family Paulowniaceae, related to and sometimes included in the family Scrophulariaceae. It is a very adaptable, extremely fast-growing woody plant, and in managed plantations, it can be harvested for saw timber in as little as 5 years. *Paulownia* can not only provide wood for a variety of products including timber, fuelwood, herbal medicines, boxes, clogs, musical instruments, and surfboards, but can also benefit the environment and ecology; for example, it can increase crop production when used for intercropping on farmland, and prevent soil erosion [28], [29]. Because of its specific characteristics and economic value, in its native China, *Paulownia* is popular for reforestation, intercropping on farmland, and roadside and garden planting. To enlarge the germplasm and breed new varieties, in recent years, several autotetraploid *paulownia* seedlings were induced successfully using colchicines [30]–[33]. The autotetraploid cultivars of *paulownia* contains two sets of the same chromosomes, and compared with their diploid progenitors, the autotetraploids have better timber quality and improved stress resistance [34], [35], which makes understanding the molecular mechanisms underlying the differences of diploids and autotetraploids imperative. To our knowledge, the different paulownia species have genetic diversity and differentiation, although miRNAs in the *P. tomentosa* [26] and *P. fortunei* species have been identified [27], there have been no detailed reports of miRNAs in *P. australis*. In the current study, we used Solexa sequencing and degradome technology to analyze four sequencing libraries constructed from the seedlings of the two *P. australis* genotypes to identify conserved and novel miRNAs and to investigate the potential roles of their transcript targets.

## Materials and Methods

### Plant culture and treatment

Plantlets of two *P. australis* genotypes (diploids, PA2; and autotetraploids, PA4) were cultured *in vitro* on medium as described previously [30], [36]. All the samples were grown at 25°C under a 16/8 h (light/dark) photoperiod for 30 days, and at least three parallel samples were prepared for each genotype. After culturing, samples were collected from nine different seedlings of each genotype and subsequently mixed, frozen immediately in liquid nitrogen, and stored at −86°C until further use.

### Construction and sequencing of the small RNA library

Total RNA was isolated from the leaves of the PA2 and PA4 samples using TRIzol reagent (Invitrogen, Carlsbad, CA, USA) following the manufacturer's instructions. Two small RNA (sRNA) libraries were constructed, one from the PA2 sample and the other from the PA4 sample, using methods described elsewhere [24], [37]. Briefly, bands in the 18–30 nt size range were purified by electrophoretic separation on a 15% denaturing polyacrylamide gel and then successively ligated with 5′ and 3′ adapters. After reverse transcription and PCR, the amplified products were subjected to Solexa sequencing (Illumina, San Diego, CA, USA) at the Beijing Genomics Institute, Shenzhen, China. The data used in this publication have been deposited in the NIH Short Read Archive database (http://www.ncbi.nlm.nih.gov/sra) with accession number SRP041440 and SRP041442 (Alias: PRJNA245384 and PRJNA245383).

### Identification of conserved and novel miRNAs in the *P. australis* libraries

Low-quality reads, poly(A) reads, oversized insertions, reads shorter than 18 nt, and adaptor contaminated reads were filtered out from the raw reads to yield the clean reads. The length distribution of the 18–30 nt clean reads was analyzed and the reads were mapped to the sequences in the *P. australis* UniGene database (http://www.ncbi.nlm.nih.gov/sra; Accession Number SRP032321) using SOAP [38]. Perfectly matched reads were analyzed by running Blastall (http://www.ncbi.nlm.nih.gov/staff/tao/URLAPI/blastall/) against the Rfam (http://www.sanger.ac.uk/software/Rfam) and GenBank databases (http://www.ncbi.nlm.nih.gov/) to discard tRNAs, rRNAs, snRNAs, snoRNAs, and other ncoRNAs. The remaining reads were annotated by aligning them against the sequences in the miRBase database 19.0 (http://www.mirbase.org/) to identify known miRNAs. When a read in the *P. australis* libraries shared homology with fewer than two mismatches with a miRNA sequence in miRBase, it was considered to be an evolutionarily conserved miRNA.

The MIREAP software (https://sourceforge.net/projects/mireap/) was used to detect novel miRNAs in the two libraries by predicting the stem-loop structures and estimating the minimal folding free energy of the unannotated sRNAs (no matches in miRBase) that mapped to the *P. australis* UniGene sequences. The stem-loop structures of the candidate precursor miRNAs (pre-miRNAs) were predicted using Mfold (http://mfold.rna.albany.edu/?q0mfold) [39]. The strict criteria used to annotate the candidate miRNAs were as described by Meyers *et al.* [40].

### Differential expression analysis of miRNAs in the two *P. australis* libraries

To identify the differentially expressed miRNAs between the PA2 and PA4 libraries, the abundance of the miRNAs in the two libraries were normalized to one million, regardless of the total number of miRNAs in each sample. Then, statistical analysis was performed based on a Poisson distribution. Finally, when the fold-change was greater than 1.0 or less than −1.0 and the *P*-values were less than 0.05, the miRNA was considered to be significantly differentially expressed. The fold-changes and *P-*values [41], [42] were calculated from the normalized expression using the following formulas:

Normalized expression  =  (actual miRNA reads/total count of clean reads) × 1,000,000

Fold-change  =  log2 (PA4 normalized reads/PA2 normalized reads)

*P*-values were calculated as,
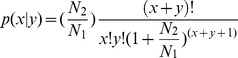


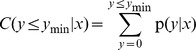


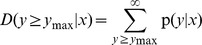



### Degradome library construction, data analysis, and target identification

Two degradome libraries were constructed from the leaves of the PA2 and PA4 plants based on a protocol published previously [14], [15]. Briefly, using T4 RNA ligase (Takara, Dalian, China), poly(A) enriched RNA was ligated to a 5′RNA adapter containing a *Mme*I recognition site. Reverse transcription was performed to generate first-strand cDNA, followed by PCR enrichment, and digestion using the *Mme*I restriction enzyme (NEB, Ipswich, MA, USA). A double-stranded DNA adapter was then ligated to the digested products using T4 DNA ligase (NEB, Ipswich, MA, USA) and gel purified for PCR amplification. The resulting PCR products were sequenced using an Illumina HiSeqTM 2000 system. Low-quality sequences and adapters were removed from the raw reads of the degradome libraries, then the remaining reads were aligned to the *P. australis* UniGene database (http://www.ncbi.nlm.nih.gov/sra; Accession Number SRP032321) using SOAP software (http://soap.genomics. org.cn/) to define the coverage rate. The PAIRFINDER software (version 2.0) [22] was used to identify the miRNA-mediated cleaved fragments. Alignments with scores not exceeding four and no mismatches at the cleavage site (between the 10th and 11th nucleotides) were considered to be the potential miRNA targets. Furthermore, t-plots were built according to the distribution of signatures (and abundances) along these transcripts to help analyze the miRNA targets and RNA degradation patterns. Based on the locations of the target genes in the *P. australis* UniGene sequences, putative target genes were selected manually and subsequently mapped to the previously identified genes that were annotated according to the annotations of their homologous sequences in the GenBank Nr and Nt databases, and in the Swiss-Prot database using BLASTX searches with an E-value cutoff of 10^−5^.

### Verification of miRNAs and their targets by quantitative real-time polymerase chain reaction

Quantitative real-time polymerase chain reaction (qRT-PCR) was used to validate and measure the expressions of selected miRNAs and their targets obtained from the Solexa sequencing and degradome analysis. The qRT-PCR was performed according to the protocol described previously [43]. For the qRT-PCR, total RNA were isolated from the leaves of two individual PA2 and PA4 plants at two developmental stages (30-day-old *in vitro* plantlets, and two-year-old saplings from field). Three biological replicates of each stage were used. The qRT-PCRs were performed in triplicate on a CFX96TM Real-Time PCR System (Bio-Rad, Hercules, CA, USA) using a SuperScript III platinum SYBR Green one-step qRT-PCR kit (Invitrogen, Carlsbad, CA, USA) according to the manufacturer's instructions. The PCR conditions were 50°C for 3 min, 95°C for 5 min, then 40 cycles of 95°C for 15 s, 55°C for 30 s, and 40°C for 10 min. Specific stem-loop primers and other primers for the miRNAs were designed based on the mature miRNA sequence. The U6 was used as an endogenous control. All the primers used for the qRT-PCR are listed in Table S1. The primers for the target genes were designed using Beacon Designer, version 7.7 (Premier Biosoft International, Ltd., Palo Alto, CA, USA), and the 18S rRNA of Paulownia was chosen as an endogenous reference gene for normalization. The relative expression level of a miRNA or target gene was calculated according to the method described previously [44].

## Results

### Analysis of sRNAs in the two *P. australis* libraries

A total of 13,895,340 and 13,537,466 raw reads were obtained in the PA2 and PA4 sRNA libraries, respectively. After removing low-quality reads, poly(A) reads, oversized insertions, reads shorter than 18-nt, and adaptor contaminated reads, we obtained 10,691,271 (PA2) and 10,712,733 (PA4) clean reads that represented 2,006,153 (PA2) and 2,418,971 (PA4) unique reads with lengths ranging from 18 to 30-nt. The majority of clean reads, 63.24% (PA2) and 68.79% (PA4), ranged from 20 to 24-nt in length, among which the 24-nt long reads were the most abundant, followed by the 21-nt long reads (Figure S1). The abundances of the 24-nt and 21-nt long sRNAs in the PA4 library were 6.48% and 0.04% more, respectively, than their abundances in the PA2 library. This atypical situation has also been reported in other hardwood species, such as *Liriodendron chinense* [45].

The clean reads were used to query the *P. australis* UniGene database (http://www.ncbi.nlm.nih.gov/sra), the non-coding RNAs sequences deposited in GenBank (http://www.ncbi.nih.gov/Genbank), the Rfam database (http://rfam.sanger.ac.uk/), and miRBase 19.0 (http://microrna.sanger.ac.uk/sequences). These searches allowed us to assign annotations to each sRNA sequence, including rRNA, tRNA, snRNA, snoRNA, and known miRNA (Table S2). As a result, 848,652 (PA2) and 931,989 (PA4) known miRNA reads were detected. Finally, 1,809,478 (PA2) and 2,308,527 (PA4) unique unannotated sRNAs remained for predicting potentially novel miRNAs.

### Identification of conserved miRNAs in *P. australis*

To detect conserved miRNAs in the two libraries, the unique reads were compared with the known miRNAs in miRBase 19.0, allowing two mismatches. In the two libraries, a total of 45 conserved miRNAs belonging to 15 miRNA families were found to share high identity with known plant miRNAs (Table 1 and Table S3). Among these 45 miRNAs, 40 and 43 were identified in PA2 and PA4, respectively; 38 of them were expressed in both libraries, two were found only in the PA2 library, and five occurred only in the PA4 library. Most of the conserved miRNAs (91.67%) were 21-nt long and the remaining miRNAs were 20 or 23-nt long (Table 1 and Table S3). This result is consistent with the current understanding that canonical miRNAs are 21-nt long, while canonical small interfering RNAs (siRNAs) are 24-nt long [46]. Among the 15 miRNA families, the miR166 family was dominant in both libraries, accounting for 89.44% (PA2) and 86.35% (PA4) of all conserved miRNA reads, followed by the miR159 family (Table 1). Several miRNA families, such as miR156, miR396, miR397, miR398, and miR482, had moderate expression levels in the two libraries, while other miRNA families showed very low levels of expression, with fewer than 100 reads, in both libraries. Moreover, different members in the same miRNA family displayed significantly different expression levels. For instance, members of the miR166 family varied in abundance from 50,932 to 563,946 reads in the two libraries (Table 1).

**Table 1:** Conserved miRNAs identified from *P. australis.*

Family	miRNA	expression		fold-change	P-value	MiRNA*expression	
		PA2	PA4	(log2PA4/PA2)		PA2	PA4
MIR169	pas-miR169a-3p	252	142	−0.83	2.27E-08	180	87
	pas-miR169b-3p	244	0	−11.16	2.77E-74	173	0
	pas-miR169c-3p	244	0	−11.16	2.77E-74	173	0
	pas-miR169d	18	26	0.53	2.35E-01	0	0
	pas-miR169e	18	26	0.53	2.35E-01	0	0
MIR159	pas-miR159-3p	117706	141387	0.26	0	115	83
	pas-miR319a-3p	1312	85	−3.95	0	16	0
MIR408	pas-miR408a-3p	1475	6263	2.08	0	50	208
	pas-miR408b-3p	1475	6263	2.08	0	50	208
MIR396	pas-miR396a	7879	6831	−0.21	1.86E-18	142	153
	pas-miR396b	2808	3441	0.29	2.11E-15	120	263
	pas-miR396c-3p	0	30	8.13	9.61E-10	13	11
	pas-miR396d-3p	0	30	8.13	9.61E-10	13	11
MIR397	pas-miR397a	676	6543	3.27	0	16	368
	pas-miR397b	678	6601	3.28	0	16	368
MIR398	pas-miR398a-3p	532	3071	2.53	0	44	1042
	pas-miR398b-3p	532	3071	2.53	0	44	1042
	pas-miR398c-3p	532	3071	2.53	0	44	1042
MIR166	pas-miR166a-3p	178621	189900	0.09	3.20E-72	1809	1883
	pas-miR166b-3p	180817	192950	0.09	1.90E-82	141	332
	pas-miR166c-3p	550961	563946	0.03	2.62E-29	1689	1755
	pas-miR166d-3p	422623	424471	0.00	2.78E-01	373	236
	pas-miR166e-3p	50932	51265	0.01	4.71E-01	490	455
MIR160	pas-miR160a	718	1445	1.01	8.76E-56	0	1
	pas-miR160b	30	40	0.41	2.38E-01	0	0
	pas-miR160c	30	40	0.41	2.38E-01	0	0
	pas-miR160d	30	40	0.41	2.38E-01	0	0
	pas-miR160e	30	40	0.41	2.38E-01	0	0
	pas-miR160f	30	40	0.41	2.38E-01	0	0
MIR156	pas-miR156a	2251	3231	0.51	5.59E-39	0	0
	pas-miR156b	2251	3221	0.51	4.75E-39	46	69
	pas-miR156c	1349	2284	0.76	1.94E-54	278	493
	pas-miR156d	1349	2284	0.76	1.94E-54	278	493
MIR164	pas-miR164	396	284	−0.48	1.51E-05	85	40
MIR167	pas-miR167	509	864	0.76	8.46E-22	18	27
MIR168	pas-miR168a	1893	2713	0.52	2.06E-33	143	236
	pas-miR168b	1895	2712	0.51	3.58E-33	137	185
MIR2118	pas-miR2118a-3p	109	136	0.32	8.78E-02	0	0
	pas-miR2118b-3p	109	136	0.32	8.78E-02	0	0
MIR482	pas-miR482a-3p	4727	6806	0.52	5.00E-83	0	0
	pas-miR482b-3p	4529	4723	0.06	5.48E-02	0	0
	pas-miR482c-3p	4728	6804	0.52	8.48E-83	0	0
MIR171	pas-miR171a	0	10	6.54	9.87E-04	0	0
	pas-miR171b	0	8	6.22	3.94E-03	0	0
	pas-miR171c	0	8	6.22	3.94E-03	0	0

### Identification of novel miRNAs in the *P. australis* sRNA libraries

The characteristic stem-loop structure of pre-miRNA was employed to predict novel miRNAs [40]. We found that 31 of the potential pre-miRNAs met this requirement; 15 pre-miRNAs exhibited the 3p:5p miRNA pair, providing more evidence that they were novel miRNAs, while the other 16 miRNAs were also considered to be potentially novel miRNAs (Table 2 and Table S4). Among the 31 candidate novel miRNAs, 10 miRNAs were common between the two libraries, while 10 and 11 were specific to PA2 and PA4, respectively. The length of the mature miRNAs varied from 20 to 23 nt, with the majority being 21-nt long. The mature miRNA sequences were localized inside the stem-loop structures, with almost half in either the 3p or 5p arms. We observed that the average pre-miRNA length was 151 nt, and these precursors had minimal folding free energies that ranged from −142.5 kcal mol^−1^ to −30.8 kcal mol^−1^ with an average of −56.3 kcal mol^−1^. In this study, compared with the abundance of the conserved miRNAs, the majority of novel miRNAs had relatively low expressions, and only five (PA2) and six (PA4) of the novel miRNA candidates had more than 1,000 reads (Table 2 and Table S4).

**Table 2:** Novel miRNAs identified from *P. australis.*

miRNA	expression		Fold-change	P-value	MiRNA* expression	
	PA2	PA4	(log2PA4/PA2)		PA2	PA4
pas-mir1	116	422	1.86	7.02E-42	106	74
pas-mir2	43	0	−8.65	1.09E-13	0	0
pas-mir3	8	3	−1.42	1.45E-01	6	5
pas-mir4a	6	0	−5.81	1.55E-02	0	0
pas-mir4b	6	0	−5.81	1.55E-02	0	0
pas-mir5a	7116	6078	−0.23	5.38E-20	208	223
pas-mir5b	7116	6078	−0.23	5.38E-20	208	223
pas-mir6a-3p	2125	4098	0.94	1.51E-139	67	71
pas-mir6b-3p	2125	4098	0.94	1.51E-139	67	71
pas-mir6c-3p	2125	4098	0.94	1.51E-139	67	71
pas-mir7-3p	696	0	−12.67	1.51E-210	6	0
pas-mir8a-3p	29	0	−8.08	4.57E-01	0	0
pas-mir8b-3p	29	11	−1.4	5.48E-02	0	0
pas-mir9	8	0	−6.23	3.87E-03	0	0
pas-mir10-3p	10	0	−6.55	9.66E-04	0	0
pas-mir11-3p	17	0	−7.31	7.49E-06	0	0
pas-mir12	5	0	−5.55	3.11E-02	0	0
pas-mir13-3p	934	1506	0.69	5.10E-31	1128	1403
pas-mir14	10	28	1.48	3.45E-03	2	1
pas-mir15a	345	0	−11.66	9.86E-105	107	0
pas-mir16a-3p	0	19	7.47	1.95E-06	0	0
pas-mir16b-3p	0	19	7.47	1.95E-06	0	0
pas-mir16c-3p	0	19	7.47	1.95E-06	0	0
pas-mir17-3p	0	8	6.22	3.94E-03	0	7
pas-mir18-3p	0	11	6.68	4.94E-04	0	0
pas-mir19-3p	0	126	10.2	1.34E-38	0	0
pas-mir20-3p	0	54	8.98	5.87E-17	0	2
pas-mir21a	0	12	6.81	2.47E-04	0	9
pas-mir21b	0	12	6.81	2.47E-04	0	9
pas-mir22-3p	0	11	6.68	4.94E-04	0	0
pas-mir23-3p	0	28	8.03	3.84E-09	0	0

### Expression patterns of conserved and novel miRNAs

The differential expression analysis was performed based on the normalized read counts for each identified miRNA. A total of 41 miRNAs showed statistically significant changes (fold change ≥1.0 or ≤−1.0, and *P*-values ≤0.05) in their relative abundance between the two libraries. Among them, 26 miRNAs were up-regulated (13 conserved miRNAs and 13 novel miRNAs; fold-change ≥1.0) and 15 were down-regulated (three conserved miRNAs and 12 novel miRNAs; fold-change ≤−1.0) in the PA4 library compared with in the PA2 library. The expression levels of some miRNAs changed significantly. For example, the expression levels of pas-miR169b-3p, pas-miR169c-3p, pas-miR396c-3p, pas-miR396d-3p, pas-miR171a, pas-miR171b, and pas-miR171c increased or decreased by about 5-fold in the PA4 library (Table 1). We also detected 21 novel miRNAs in the PA4 library that had 5-fold greater or lesser expression levels than in the PA2 library (Table 2).

### Identification of miRNAs transcript targets in *P. australis* by using degradome analysis

**Figure 1:**
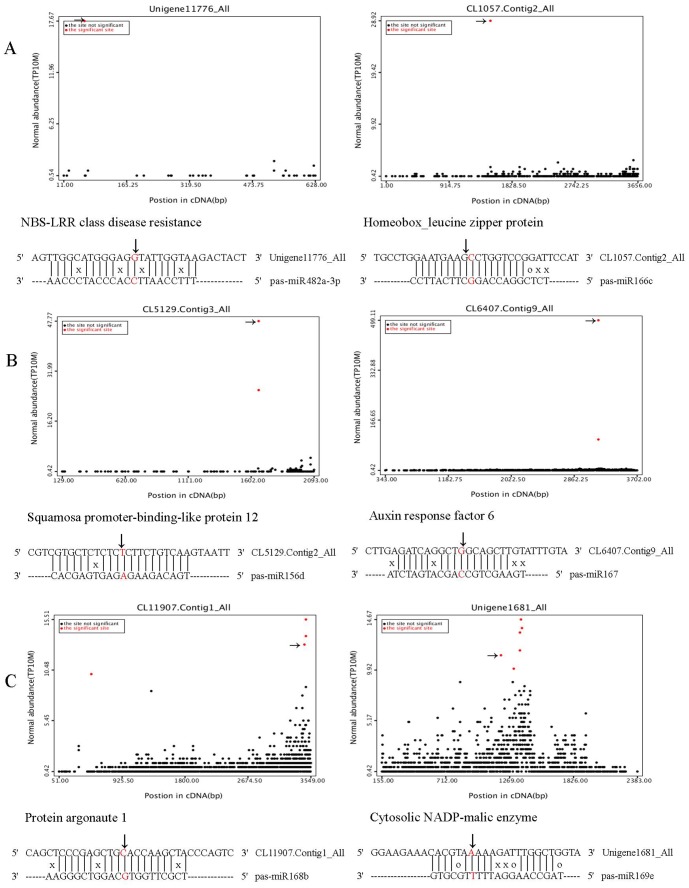
Target plots (t-plots) of miRNA targets in different categories confirmed by degradome sequencing. (A) T-plot (top) and miRNA: mRNA alignments (bottom) for two category I targets, Unigene11776_All and CL1057.Contig2_All transcripts. The arrow indicates signatures consistent with miRNA-directed cleavage. The solid lines and dot in miRNA: mRNA alignments indicate matched RNA base pairs and GU mismatch, respectively, and the red letter indicates the cleavage site. (B) CL5129.Contig3_All and CL6407.Contig9_All, a category II target for pas-miR156d and pas-miR167. (C) CL11907.Contig1_All and Unigene1681_All, a category III target for pas-miR168b and pas-miR169e.

**Figure 2:**
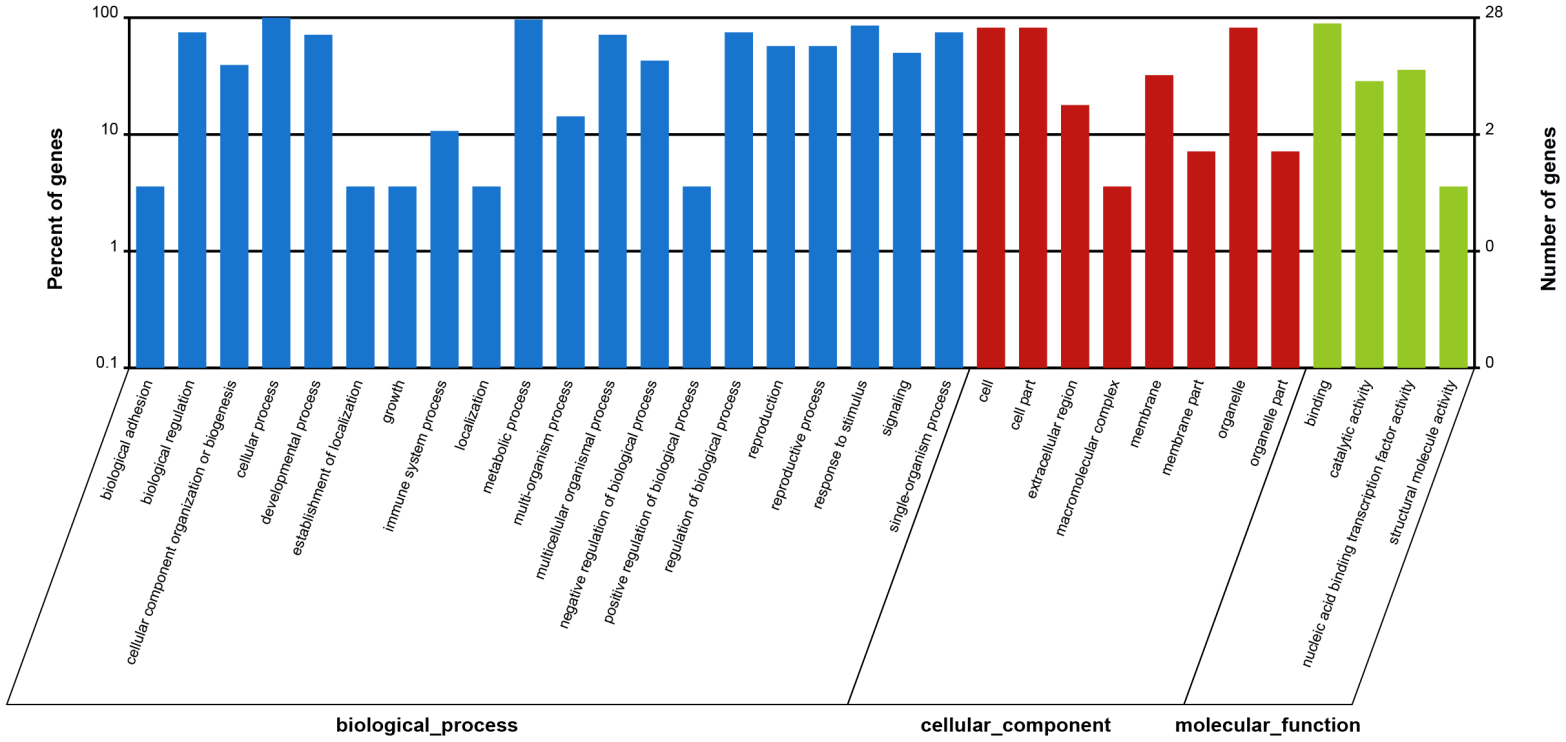
Gene Ontology analysis of miRNA targets in *P. australis*.

To better understand the physiological functions and biology processes that these miRNAs may be involved in during the development of *P. australis*, transcript target identification was performed based on the degradome approach. Using PAIRFINDER, a total of 53 targets were predicted to be cleaved by 11 of the conserved miRNA families and three novel miRNA candidates (Table S5). The target transcripts were pooled and grouped into three categories (I–III) according to their relative abundances [14], [15] (Figure 1). Among these identified targets, 32 (37 cleavage sites) belonged to category I, and 20 (34 cleavage sites) and six (six cleavage sites) targets belonged to categories II and III, respectively (Table S5). We then performed a Gene Ontology (GO) analysis to assign functional annotations to the predicted target genes, as described previously [47]. More than 80% of the target genes were annotated as being involved in regulation of biological processes, and the GO annotations for the predicted target genes are shown in Figure 2. The Kyoto Encyclopedia of Genes and Genomes (KEGG) Pathway database was used to further classify the miRNA target genes. The KEGG Pathway annotation showed that the target genes were involved in metabolic pathways, plant-pathogen interaction, pyruvate metabolism, carbon fixation in photosynthetic organisms, purine metabolism, RNA polymerase, pyrimidine metabolism, nitrogen metabolism, plant hormone signal transduction, cellular metabolism, and disease. (Table S5). The prediction and annotation of the miRNA target genes may provide some new insights into how *P. australis* miRNAs regulate gene expression in this plant.

### Validation of the candidate miRNAs and their targets by qRT-PCR

**Figure 3:**
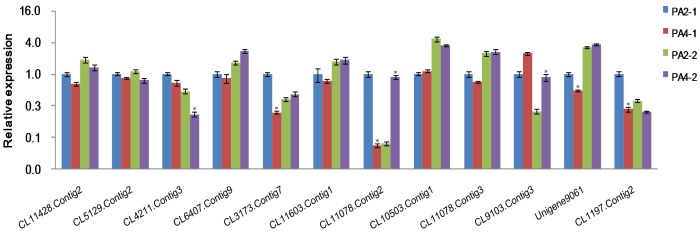
Relative expression levels of the target genes in *P. australis*. PA2-1, 30-day-old diploid in vitro plantlets; PA4-1, 30-day-old autotetraploid in vitro plantlets; PA2-2, two-year-old diploid saplings; PA4-2, two-year-old autotetraploid saplings. CL11428.Contig2 (SPL6) and CL5129.Contig2 (SPL12) targeted by pas-miR156c, CL3173.Contig7 (ARF10) and CL11603.Contig1 (ARF18) targeted by pas-miR160a, CL4211.Contig3 (ARF8) and CL6407.Contig9 (ARF6) targeted by pas-miR167, CL11078.Contig2 (SCL6), CL10503.Contig1 (SCL15) and CL11078.Contig3 (SCL22) targeted by pas-miR171a, CL9103.Contig3 (Transcription factor TCP4) targeted by pas-miR319a-3p; Unigene9061 (Serine/threonine protein kinase) targeted by pas-mir1; CL1197.Contig2 (Zinc finger CCCH domain-containing protein 53) targeted by pas-mir22-3p. Three independent biological replicates were performed. Values are means ± SD (n = 3).The expression levels of targets were normalized to 18SrRNA. The normalized miRNA levels in the PA2-1 were arbitrarily set to 1. *: Statistically significant differences between PA2 and PA4 under the same developmental stages (p-value was less than 0.05)

To verify the existence and expression levels of the miRNAs determined from the high-throughput Solexa sequencing, 12 miRNAs with different expression levels were randomly selected for qRT-PCR analysis. As shown in Figure S2, the expression patterns of the miRNAs obtained by qRT-PCR showed similar trends to their expression patterns in the two libraries as determined by Solexa sequencing. Compared with their expressions in PA2, the expression levels of pas-miR156c, pas-miR398a-3p, pas-miR408a-5p, and pas-mir22-3p were up-regulated in PA4 in the 30-day plantlets and in the 2-year saplings, pas-miR319a-3p were on the contrary. Further, the expressions of pas-miR160a, pas-miR167, pas-miR171a, pas-miR397a, pas-mir1, and pas-mir14 in PA4 were up-regulated in the 30-day plantlets, and down-regulated in the PA4 in the 2-year saplings, while the expressions of pas-mir3 were the opposite to this. Thus, with the development of the plants, the expression levels of some of the miRNAs showed different trends. Six miRNAs (pas-miR160a, pas-miR167, pas-miR319a-3p, pas-miR398a,-3p pas-miR408a-3p, pas-mir1) had the same expression trend between PA2 and PA4 in the two stages. Furthermore, to confirm the reliability of degradome sequencing technology and the potential correlation between miRNAs and their transcript targets, 12 genes from two *P. australis* genotypes were also selected for qRT-PCR assays. The results showed that except the targets of auxin response factor (ARF) ARF8 (CL4211.Contig3) and scarecrow-like protein (SCL) SCL15 (CL10503.Contig1), the expression levels of the rest targets were inversely correlated with these of the corresponding miRNAs (Figure 3). During the different developmental stages, pas-miR160a, pas-miR167, pas-miR171 and pas-mir1 at a relatively higher level in the PA4 than in the PA2 at the 30-day plantlets stage, and lower level at the 2-year saplings stage, while its transcript targets, CL3173.Contig7, CL11603.Contig1, CL6407.Contig9, CL11078.Contig2, CL11078.Contig3, and Unigene9061 expressed in the reverse way as expected, and these coding for proteins are members of the auxin response factors ARF10, ARF18 and ARF6, the scarecrow-like proteins SCL6 and SCL22, and serine/threonine protein kinase, respectively. The expression levels of pas-miR319a-3p in PA4 were significantly lower than in the PA2 at two treatment stages, while the reverse was true for the transcription factor TCP4 (CL9103.Contig3) (Figure 3). Moreover, a reverse trend was noted between pas-miR156c and its target genes coding for squamosa promoter-binding-like protein (SPL) SPL6 and SPL12 (CL11428.Contig2 and CL5129.Contig2), and between pas-mir22-3p and its target gene coding for zinc finger CCCH domain-containing protein 53 (CL1197.Contig2) in the PA4 as compared to the PA2 (Figure 3). These results indicated that the miRNA and transcript target expression patterns were very complex and varied during the growth and development of *P. australis*. Furthermore, the possible roles of these miRNAs in the genome duplication changes from diploid to autotetraploid in these plants were revealed.

## Discussion

Variations in plant morphology and physiology resulting from genome duplication have occurred in many plants, such as *Triticum*, *Gossypium*, *Spartina*, *Tragopogon*, *Brassica* and *Solanum* [48], [49]. Generally speaking, genome duplication has led to the production of fast-growing, high-quality plants [50], [51]. The autotetraploid *P. australis*, which contains two sets of the same chromosomes, displayed apparent alterations in morphology, growth development, physiology, and gene expression when compared with their diploid counterparts [34], [35]. However, the mechanisms for these changes are poorly understood. MiRNAs are a class of endogenous small RNAs that have been involved in many processes, including growth, development, and resistance to stress and disease, by their ability to regulate gene expression in plants [52]–[54]. To understand the functions of miRNAs in diploid and autotetraploid *P. australis,* in this present study, we used Solexa sequencing and degradome approaches to construct two sRNA libraries and two degradome libraries from the PA2 and PA4 plants to identify conserved and novel miRNAs and their transcript targets. A total of 45 conserved miRNAs belonging to 15 miRNA families and 31 potential novel miRNA candidates along with 53 transcript targets were identified across the PA2 and PA4 libraries. Most of the identified conserved miRNA families are also conserved in other plant species, including *Populus tomentosa* [53], *Populus euphratica* [55], *Oryza sativa* [3], and *Arabidopsis thaliana* [56]. In the PA2 and PA4 libraries, the expression patterns varied dramatically among the different miRNA families, and different members in the same miRNA family also displayed significantly different expression levels. For instance, the read number varied from 26 (miR171 family) to 1,422,532 (miR166 family) (Table 1), and members of the miR166 family varied in abundance from 50,932 to 563,946 reads (Table 1). Moreover, the majority of novel miRNAs had relatively low expressions, and only five (PA2) and six (PA4) of the novel miRNA candidates had more than 1,000 reads (Table 2). Our results are in accordance with previous reports that novel miRNAs were often represented in relatively lower levels than conserved miRNAs [57], [58]. Furthermore, it is possible that the low-expression novel miRNAs may play particular functions in specific tissues, during developmental stages, or under various growth conditions. Whether these low-expression miRNAs are expressed at higher levels in other tissues or developmental stages, or are regulated by environmental stresses, remain to be investigated.

Previous studies have shown that many of the genes appeared to be methylated in tetraploid Paulownia plants specifically after genome duplication by the methylation-sensitive amplified polymorphism analysis [59]. DNA methylation has been reported to be involved in inducing gene silencing, which can restart or change the genes expression levels [60]. Salmon et al. [61] found that significant changes in DNA methylation patterns could explain the morphological plasticity and larger ecological amplitude of Spartina allopolyploids. Indeed, we found that the expression levels of many of the differentially expressed miRNAs in the PA4 library were not increased by more than two-fold compared with their expressions in the PA2 library. However, the expressions of about half of these miRNAs were significantly different in the two libraries. Some of the miRNAs were expressed at similar level in the PA4 and PA2 libraries (Table 2). These findings are similar to those reported previously in *P. tomentosa, P. fortunei* and tetraploid *Arabidopsis thaliana* lines [26], [27], [62], suggesting that the genome merger in the PA4 plants lead to nonadditive expression of the miRNA primary transcripts and miRNA target genes. Furthermore, the expression patterns of the differentially expressed miRNAs and transcript targets at different development stages were validated by qRT-PCR. The result showed that the differentially expressed miRNAs caused different expression levels in their transcript targets. Interestingly, we also found the expression levels of two target genes (CL4211.Contig3 and CL10503.Contig1) were inconsistent with those of their corresponding miRNAs (pas-miR167 and pas-miR171a). The similar phenomena were also observed in *P. tomentosa*, *Phalaenopsis Aphrodite* and cotton [26], [63], [64], indicating that the other mechanisms of regulating expression of the target genes exist. Above all, these results imply that the miRNAs with significantly varied expressions in the PA2 and PA4 *P. australis* plants are probably involved in the epigenetic changes of PA4 plants; however, the relation between the miRNA expression patterns and genome duplication may be more complex than we first thought.

To understand the biological functions of miRNAs, it is necessary to identify their transcript targets. In the present study, to avoid false-positive predictions of miRNA transcript targets in *P. australis*, we identified the 53 transcript targets for 11 miRNA families and three novel miRNA candidates by degradome sequencing, which opens up a new avenue for high-throughput validation of splicing targets [14], [15]. The target genes predicted for the conserved miRNAs in *P. australis* were similar or functionally related to validated plant miRNA targets, which were annotated as being involved in diverse physiological processes. For instance, pas-miR156 targeted the SPL protein family, which can affect diverse developmental processes such as leaf development, shoot maturation, phase change, and flowering in plants [65]; and pas-miR167 targeted ARF6 and ARF 8, which belong to a class of transcription factors known to control multiple processes in plants, including the regulation of gynoecium and stamen maturation, and seed dispersal [66], [67]. Thus, our results support the idea that conserved miRNAs take part in essential physiological processes in plants.

The analysis of the target genes identified for the differentially expressed miRNAs revealed that some of the target genes may play important roles in plant morphology and physiology. We found that the expression level of pas-miR319a-3p decreased by about 3-fold in PA4 compared with its level in PA2, and pas-miR319a-3p was predicted to target TCP transcription factors, which are plant-specific transcription factors that have been shown to participate in specifying plant morphological traits, such as organ border delimitation [68], cell division and proliferation [69], flower and leaf shape, and shoot outgrowth [70]. In *Arabidopsis*, the TCP transcription factors have been related to control of the morphology of shoot lateral organs and formation of the shoot meristem-dependent regulation of the expression of boundary-specific genes [71]. The putative transcription factor SCL is a number of GRAS protein family that are involved in several aspects of plant growth and development, including control of asymmetric cell division, maintenance of stem cell status, and induction of the regeneration of the root tip after laser ablation, and SCL expression has been associated with auxin distribution in root apical meristem [72]–[74]. In this study, three SCL genes were predicted to be targeted by the miR171 family, which was up-regulated in the PA4 plants. This result suggested that SCL may be involved in the formation of adventitious root and the other aerial organs of *Paulownia*. L-type lectin-domain containing receptor kinases (lecRKs) and zinc finger CCCH domain-containing protein were predicted to be targeted by pas-miR1 and pas-miR22-3p, respectively, suggesting that, besides their possible involvement in plant development, some of the miRNA target genes identified in this study could play fundamental roles in biotic and abiotic stresses. A previous study showed that LecRKs were most likely plasma membrane proteins, and were probably involved in mediating protein-protein interactions with a wide range of functions such as recognition of oligosaccharide or lipochitooligosaccharide signals, linking ABA-signaling, and response to salt, drought, cold stress, wounding, and disease in plants [75], [76]. Zinc finger CCCH domain-containing protein is a kind of RNA-binding protein and many studies have shown that it may be regulated by abiotic or biotic stresses, and could have regulatory functions in mRNA processing [77]–[80], thus supporting the possible roles for pas-miR1 and pas-miR22-3p in the adaptive response of PA4 to abiotic stress.

Based on the GO analysis of the targets of the identified miRNAs in the two *P. australis* genotypes plants, the target genes were separated into the biological process, cellular component, and molecular function. Some of the genes were annotated as being involved in biological regulation, cellular process, developmental process, response to stimulus, metabolic process, cell, cell part, organelle components, binding, and catalytic activity (Figure 2). Thus, our results suggest that the transcript targets might be closely related to the observed differences of phenotype (environmental adaptations) and resistance to biotic and abiotic stresses between the PA4 and PA2 plants. The functional role of the differentially expressed miRNAs will be the focus of future investigations. No target genes were predicted for many of the miRNAs identified in the two *P. australis* sRNA libraries, and a few of the predicted target genes were annotated as being of unknown function and hypothetical genes. Careful analysis of these potential targets will contribute further to our understanding of the role of miRNAs in *P. australis*.

In summary, miRNA target genes were identified using a degradome approach that included functional annotation and pathway analyses based on the GO and KEGG databases. Some of the transcript targets regulated by the differentially expressed miRNAs were related to the physiology and environmental adaptations. Our results suggest that the significantly varied expression miRNAs in the two *P. australis* genotypes are probably involved in the epigenetic changes of PA4 plants, the correlation between the miRNA expression pattern and genome duplication may be more complex than we first thought. Taken together, this study provides beneficial information for elucidating the miRNA-mediated regulation of transcript targets in *P. australis* and related species.

## Supporting Information

Figure S1 **Length distribution of sRNAs in** ***P. australis*** **.** (A) Size distribution of total sequences. (B) Size distribution of unique sequences.
(TIF)

Figure S2 **Results from qRT-PCR of miRNAs in** ***P. australis.*** PA2-1, 30-day-old diploid in vitro plantlets; PA4-1, 30-day-old autotetraploid in vitro plantlets; PA2-2, two-year-old diploid saplings; PA4-2, two-year-old autotetraploid saplings. Three independent biological replicates were performed. Values are means ± SD (n = 3). The expression levels of miRNAs were normalized to U6. The normalized miRNA levels in the PA2-1 were arbitrarily set to 1. *: Statistically significant differences between PA2 and PA4 under the same developmental stages (p-value was less than 0.05).
(TIF)

Table S1 **Quantitative RT-PCR validated miRNAs and their targets primers.**
(XLS)

Table S2 **Categories and statistical summary of sRNAs in** ***P. australis*** **.**
(DOCX)

Table S3 **The sequences of conserved miRNAs identified from** ***P. australis*** **.**
(XLSX)

Table S4 **The sequences of novel miRNAs identified from** ***P. australis*** **.**
(XLSX)

Table S5 **Identified targets of miRNAs involved in** ***P. australis*** **by degradome analysis.**
(XLSX)
