# Supplementary figures and images for: Transcriptome/Degradome-Wide Discovery of MicroRNAs and Transcript Targets in Two Paulownia australis Genotypes
Source: PLoS One. 2014 Sep 8;9(9):e106736. doi: 10.1371/journal.pone.0106736 (PMC4157796; doi:10.1371/journal.pone.0106736)

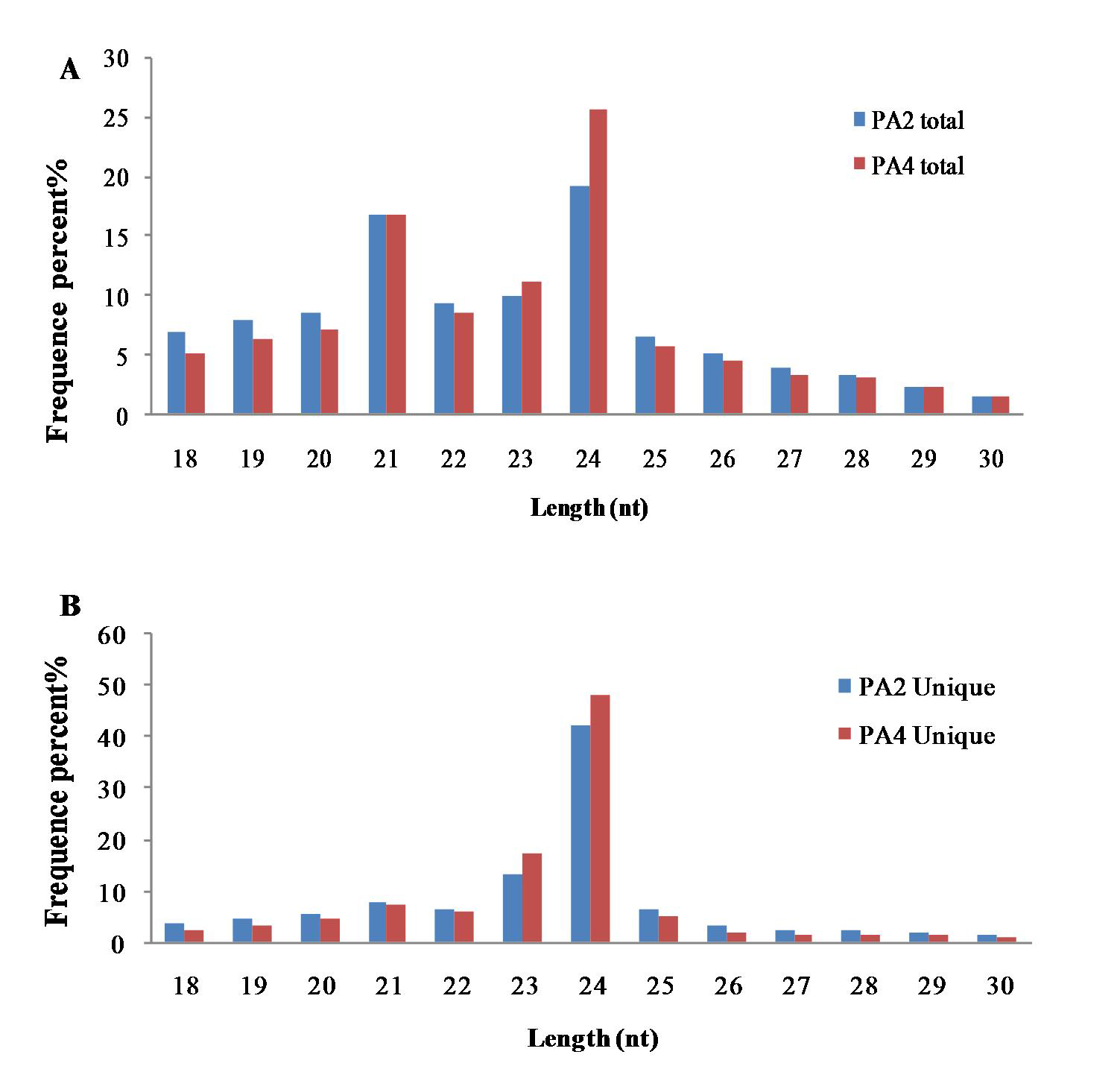

Supplement: Figure S1 — Length distribution of sRNAs in P. australis . (A) Size distribution of total sequences. (B) Size distribution of unique sequences. (TIF) [file pone.0106736.s001.tif]

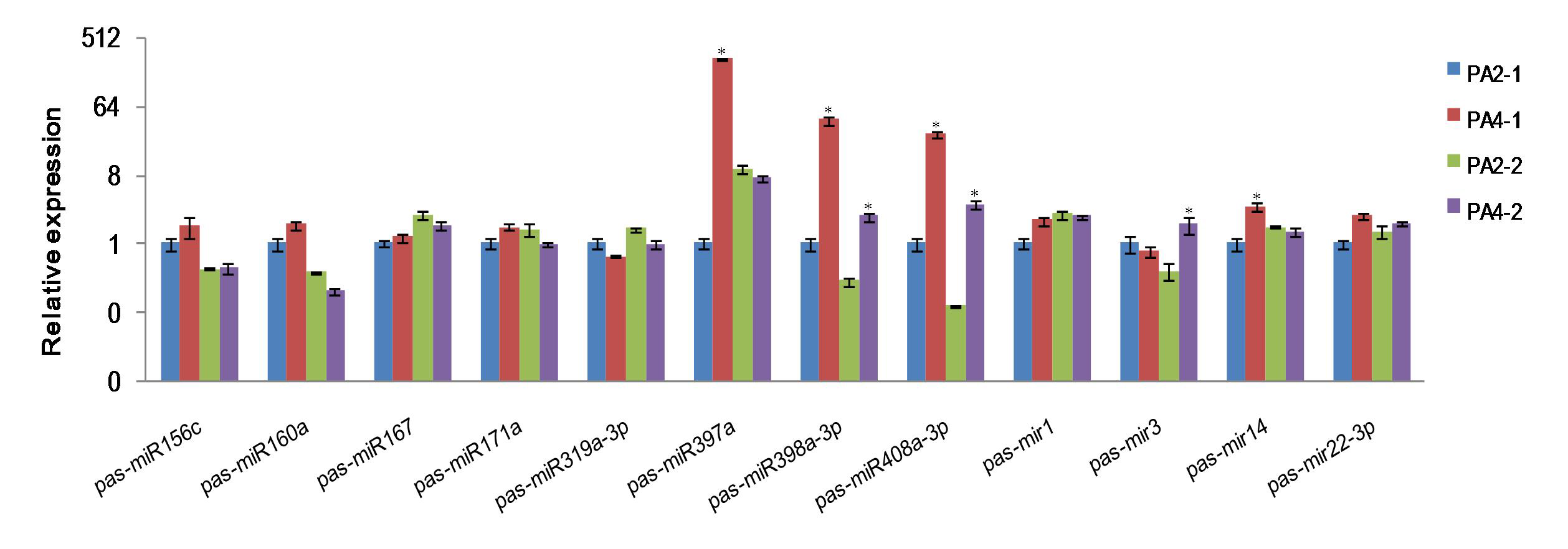

Supplement: Figure S2 — Results from qRT-PCR of miRNAs in P. australis. PA2-1, 30-day-old diploid in vitro plantlets; PA4-1, 30-day-old autotetraploid in vitro plantlets; PA2-2, two-year-old diploid saplings; PA4-2, two-year-old autotetraploid saplings. Three independent biological replicates were performed. Values are means ± SD (n = 3). The expression levels of miRNAs were normalized to U6. The normalized miRNA levels in the PA2-1 were arbitrarily set to 1. *: Statistically significant differences between PA2 and PA4 under the same developmental stages (p-value was less than 0.05). (TIF) [file pone.0106736.s002.tif]
