# Supplementary material for: Transcriptome/Degradome-Wide Discovery of MicroRNAs and Transcript Targets in Two Paulownia australis Genotypes
Source: PLoS One. 2014 Sep 8;9(9):e106736. doi: 10.1371/journal.pone.0106736 (PMC4157796; doi:10.1371/journal.pone.0106736)
Supplement: Table S2 — Categories and statistical summary of sRNAs in P. australis . (DOCX) [file pone.0106736.s004.docx]

| **category** | **PA2** | | | | **PA4** | | | |
| --- | --- | --- | --- | --- | --- | --- | --- | --- |
|  | **Unique sRNAs** | **Percent%** | **Total**  **sRNAs** | **Percent%** | **Unique**  **sRNAs** | **Percent%** | **Total**  **sRNAs** | **Percent%** |
| total | 2,006,153 | 100 | 10,691,271 | 100 | 2,418,971 | 100 | 10,712,733 | 100 |
| miRNA | 1,405 | 0.07 | 848,652 | 7.94 | 1,372 | 0.06 | 931,989 | 8.70 |
| rRNA | 172,573 | 8.60 | 3,705,229 | 34.66 | 90,943 | 3.76 | 3,029,195 | 28.28 |
| snRNA | 2,678 | 0.13 | 11,122 | 0.1 0 | 2,756 | 0.11 | 10,667 | 0.10 |
| snoRNA | 1,980 | 0.10 | 24,791 | 0.23 | 1,685 | 0.07 | 23,106 | 0.22 |
| tRNA | 18,039 | 0.90 | 231,514 | 2.12 | 13,688 | 0.57 | 243,457 | 2.27 |
| unannote | 1,809,478 | 90.20 | 5,875,196 | 54.95 | 2,308,527 | 95.43 | 6,474,319 | 60.44 |

**Table S2 Categories and statistical summary of sRNAs in *P. australis***
